# ﻿Diversity of ectoparasitic bat flies (Diptera, Hippoboscoidea) in inter-Andean valleys: evaluating interactions in the largest inter-Andean basin of Colombia

**DOI:** 10.3897/zookeys.1221.127890

**Published:** 2024-12-31

**Authors:** Camila López-Rivera, Laura Natalia Robayo-Sánchez, Alejandro Ramírez-Hernández, Jerson Andrés Cuéllar-Saénz, Juan Diego Villar, Jesús Alfredo Cortés-Vecino, Fredy A. Rivera-Páez, Paula Andrea Ossa-López, Erika M. Ospina-Pérez, Jose J. Henao-Osorio, Alexandra Cardona-Giraldo, Javier Racero-Casarrubia, Miguel E. Rodríguez-Posada, Darwin M. Morales-Martinez, Marylin Hidalgo, Héctor E. Ramírez-Chaves

**Affiliations:** 1 Grupo de Investigación GEBIOME, Departamento de Ciencias Biológicas, Facultad de Ciencias Exactas y Naturales, Universidad de Caldas, Calle 65 No. 26-10, 170004, Manizales, Caldas, Colombia Universidad de Caldas Manizales Colombia; 2 Grupo de Investigación Parasitología Veterinaria, Laboratorio de Parasitología Veterinaria, Facultad de Medicina Veterinaria y de Zootecnia, Universidad Nacional de Colombia, Carrera 30 No. 45-03, 111321, Bogotá D.C., Colombia Universidad Nacional de Colombia Bogotá Colombia; 3 Grupo Epidemiología y Salud Pública, Universidad de La Salle, Bogotá D. C., Colombia Universidad de la Salle Bogotá Colombia; 4 Grupo Enfermedades Infecciosas, Departamento de Microbiología, Pontificia Universidad Javeriana, Bogotá D.C., Colombia Pontificia Universidad Javeriana Bogotá Colombia; 5 Grupo de Investigación Biodiversidad Unicórdoba, Facultad de Ciencias Básicas, Universidad de Córdoba, Montería, Córdoba, Colombia Universidad de Caldas Manizalez Colombia; 6 Fundación Reserva Natural La Palmita, Centro de Investigación, Grupo de investigaciones territoriales para el uso y conservación de la biodiversidad, Bogotá, Colombia Universidad de Córdoba Córdoba Colombia; 7 Museum of Natural Science and Department of Biological Sciences, Louisiana State University, 119 Foster Hall 70803, Baton Rouge, Louisiana, USA Fundación Reserva Natural La Palmita, Centro de Investigación Bogotá Colombia; 8 Centro de Museos, Museo de Historia Natural, Universidad de Caldas, Manizales, Colombia Louisiana State University Louisiana United States of America

**Keywords:** Chiroptera, Dry Forest, Magdalena River, Nycteribiidae, specialization, Streblidae

## Abstract

Flies belonging to the families Streblidae and Nycteribiidae are highly specialized arthropods that feed on the blood of bats. Their morphology varies and has adapted throughout their coevolutionary history with hosts. Bat flies are often associated with specific bat species and can establish distinct infracommunities. Interaction networks have been used to better understand these associations, revealing interaction modules between bats and their parasites. The Magdalena River basin is the largest in Colombia, encompassing a wide variety of climatic and ecological conditions, with up to 98 bat species reported. We conducted field trips to capture bats and bat flies in different locations along the basin and reviewed literature records and biological collections to gather additional data on interactions between bats and bat flies in this region. We found a high diversity of bats and bat flies in the Magdalena River basin, revealing a medium specialization and modularity in these interactions. We identified bat fly infracommunities and negative associations between certain bat fly species, suggesting competition for resources within hosts. The specialization is similar to that reported in degraded and fragmented habitats where the availability of shelters decreases, favoring the overcrowding of bats, forming multi-species colonies. In conclusion, our study provides important information on the interactions between bats and bat flies in the Magdalena River basin, expanding knowledge about the diversity and structure of these communities in inter-Andean landscapes.

## ﻿Introduction

Ectoparasitic flies of the families Streblidae and Nycteribiidae (Diptera: Hippoboscoidea) are highly specialized hematophagous arthropods associated with bats (Wenzel et al. 1966; Marshall 1982). Currently, Nycteribiidae are considered monophyletic, while Streblidae are paraphyletic with the New World Streblidae placed apart from all Old-World taxa (Dittmar et al. 2006; 2015). The morphology of bat flies’ species within Streblidae and Nycteribiidae is highly variable (Dick and Patterson 2006). Species within Nycteribiidae have dorsoventrally flattened bodies, but also have reduced eyes and all species are apterous (Dick and Patterson 2006). In contrast, species of Streblidae can have laterally compressed, dorsoventrally flattened or uncompressed bodies, reduced compound eyes, and the wings may be normal, reduced, or absent (Dick and Patterson 2006; Dick and Miller 2010; Dick and Dittmar 2014).

The morphological adaptations of bat flies can be attributed in part to coevolutionary history with their hosts (Poinar and Brown 2012). Most bat flies are monoxenes (host-specific), but others may be oligoxenes (associated with more than one species of the same genus), pleioxenes (associated with more than one species of the same subfamily or family), and to a lesser extent, polyoxenes (associated with different species of different families) (Wenzel et al. 1966; Dick 2005; Dick and Gettinger 2005; Dick and Miller 2010). Similarly, bats may have associations with a limited number of coexisting but spatially segregated morphologically distinct bat flies, which can form “infracommunities” (ter Hofstede et al. 2004; Dick 2005; ter Hofstede and Fenton 2005; Dick and Patterson 2006). Based on the region of the host body where the bat flies are typically found, there are three ecomorphological groups: (1) wing crawlers, which include flies that predominantly inhabit the wing membrane; (2) fur runners, which are flies that particularly have long hind legs and are found mainly on the hairy body, moving on the surface of the fur; and (3) fur swimmers, which include species characterized by a compressed head and body, usually possessing ctenidia, adapted to navigate through the fur of the host (ter Hofstede et al. 2004; Dick 2005).

The study of host-parasite interactions is critical to uncovering ecological and coevolutive patterns and processes, and is key in the study of emerging infectious diseases (Swann et al. 2015). Ecologically, interaction networks reveal modules or interaction groups that are formed when bats share the same species of ectoparasites (Grilli et al. 2016), providing insight into the structure and interconnectedness of host and ectoparasite assemblages (Blüthgen et al. 2006). Also, interaction networks can determine the ecological role of each species and the complexity of their interactions (Lindeman 1942; Pilosof et al. 2017). In terms of coevolution, the interaction studies can determine how closely related the parasitic species are in the phylogeny or whether these species share ecological traits (Patterson et al. 2008; Zarazúa-Carbajal et al. 2016; Durán et al. 2018; Hernández-Martínez et al. 2018).

Despite bat flies being generally distributed globally due to the wide range of their bat hosts, the tropics exhibit greater species richness and endemism (Guerrero 1994; Dittmar et al. 2015). This phenomenon is often associated with the high diversity of bat species in tropical areas (Hutson et al. 2001). Unfortunately, knowledge of bat fly-bat interactions has been restricted to fragmented records at a local scale (Zapata-Mesa et al. 2024). For example, in the Neotropics, Colombia is home to one of the highest bat species diversity globally with 222 species (Ramírez-Chaves et al. 2021). Nevertheless, the richness of bat flies in the country is underestimated. Colombia has records of 81 species of Streblidae and 11 of Nycteribiidae (Dick et al. 2016; Graciolli et al. 2016; Pastrana-Montiel et al. 2019; Wolff et al. 2023), which is a lower diversity compared with neighboring countries such as Brazil, which has 181 bat species (Garbino et al. 2022), with 84 species of Streblidae and 26 Nycteribiidae, respectively (Graciolli 2018). A similar situation occurs in Venezuela, where 172 bat species have been reported (Delgado-Jaramillo et al. 2016), with 121 species of Streblidae and 10 of Nycteribiidae, respectively (Bezerra et al. 2016; Laurenço et al. 2016).

Extensive research on bat flies in Colombia has spanned more than four decades (Marinkelle and Grose 1981; Herrera-Sepúlveda 2013; Durán et al. 2018; Calonge-Camargo and Pérez-Torres 2018; Liévano-Romero et al. 2019), but numerous information gaps persist regarding the presence, diversity, distribution, and ecological interactions of these ectoparasitic flies (Durán et al. 2018; Liévano-Romero et al. 2019). Recent studies have provided new insights into bat-fly interactions in various natural regions of the country, such as the Orinoquia (Liévano-Romero et al. 2019; López Rivera et al. 2022; Ospina-Pérez et al. 2023), the Caribbean (Durán et al. 2018; Calonge-Camargo and Pérez-Torres 2018), and the Andes (Tamsitt and Fox 1970; Tarquino-Carbonell et al. 2015; Ascuntar-Osnas et al. 2020; Raigosa-Álvarez et al. 2020). However, there remains a gap in knowledge in the Andean region and the inter-Andean basins such as the one formed by the Magdalena River.

The inter-Andean basin of the Magdalena River covers 257,000 km^2^ and re­presents 24% of Colombia’s continental territory (Restrepo and Syvitski 2006). This basin is critical for host-parasite interaction studies for many reasons. First, the Magdalena River basin harbors a rich bat fauna with nearly 98 bat species (IAvH 2021), representing nearly 45% of the country’s bat species; however, there is limited information regarding the ectoparasitic flies that coexist with them. Second, the Magdalena River basin has more than 30 million inhabitants, around 79% of the country’s population (Restrepo and Syvitski 2006). Third, the basin has undergone significant deforestation being one of the areas with most dramatic forest reduction in Colombia between the years 1970 and 2000 (Etter et al. 2008). Considering the last two events, a dense human population and high deforestation rates, the Magdalena River basin is an ideal region for studying of the effects of habitat degradation of the prevalence of parasites, and on the emerging infectious diseases, especially in animals considered as vectors, such as bats and their parasites. In this context, this study seeks to unveil the extent of species richness within Streblidae and Nycteribiidae to elucidate the complex interactions with bats in the main inter-Andean basin of Colombia.

## ﻿Materials and methods

### ﻿Study area

The Magdalena River forms the largest inter-Andean basin of Colombia covering 257,438 km^2^ of national territory. It originates at the head of the Colombian Massif at an elevation of 3,865 m in the Puracé National Natural Park and flows into the Caribbean Sea in Bocas de Ceniza in the Department of Atlántico. This basin exhibits a great diversity of geological, edaphic, climatic, hydraulic, sedimentological, and morpho-dynamic conditions, forming a highly complex socio-ecological system (Gallo-Vélez et al. 2023). It crosses 1,540 km from south to north along 13 departments of Colombia, where 79% of the country’s population resides, making it an area of economic importance since 80% of Colombia’s GDP is generated there. The Magdalena River basin is significantly influenced by human activity, including deforestation, poor soil conservation and mining practices (Restrepo and Syvitski 2006).

Due to its geographical location, the climate of the region is tropical, primarily determined by altimetric variations, the relief topography and the influence of the Intertropical Confluence Zone, which generates two wet and two dry periods that occur interspersed throughout the year (León et al. 2000). Other factors that influence the climatic characteristics of the Magdalena River basin are precipitation, temperature, relative humidity, sunlight, and wind, which can create microclimates around the basin (IDEAM 2001; Nardini et al. 2020). According to Holdridge (1978), the Magdalena River basin encompasses five altitudinal zones: the tropical zone (0–1,000 m), the premontane zone (1,000–2,000 m), the submontane zone (2,000–3,000 m), and the Andean zone (3,000–4,000 m). The Magdalena River Basin supports ecosystems of Andean forests (26.36%), paramo (1.96%), xerophytic vegetation (3.01%), and wetlands (2.56%).

### ﻿Field trips

**Figure 1.**
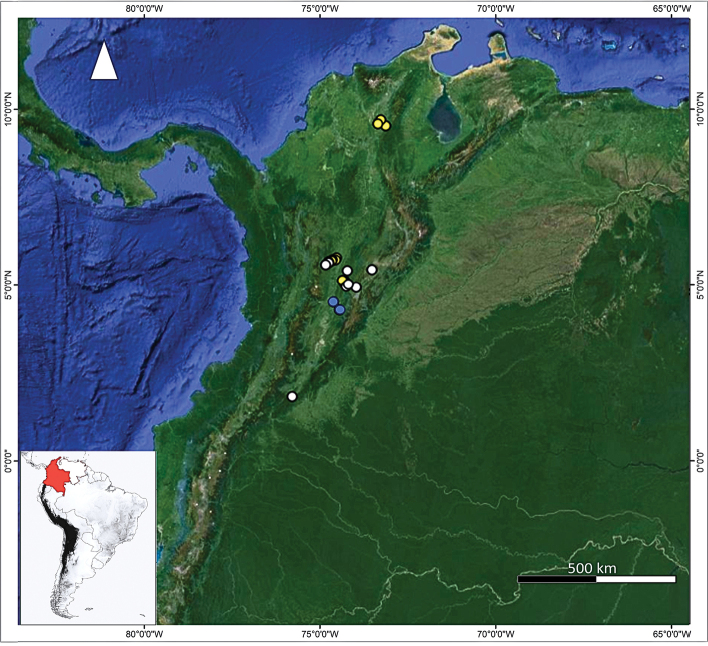
Locality records of bat flies (Nycteribiidae and Streblidae) in the inter-Andean Magdalena River basin, Colombia. Yellow circles indicate localities where field trips were conducted, blue circles are localities reported in the literature, and white circles indicate records of specimens in the ectoparasite collection of the Museo de Historia Natural de la Universidad de Caldas (MHN-UCa-Ec). The Magdalena River basin is indicated in green.

We conducted field trips in 12 localities in the Magdalena River basin between March, July and September 2021, April and November 2022, and January and March 2023. Specific dates for each locality are shown in Table 1. Four sampling sites were in the Department of Caldas at elevations between 170 and 650 m (Table 1). Five localities were in the Department of Cundinamarca, with elevations between 800 and 1,900 m. Three localities were within the Department of Cesar, with elevations between 50 and 200 m (Table 1; Fig. 1).

**Table 1.** Sampling localities (1–14) of bats and bat flies between 2021 and 2023 in the departments of Caldas, Cesar and Cundinamarca in the Magdalena River basin in Colombia. Localities obtained from the literature (15–16), and from specimens housed at the ectoparasite collection (MHN-UCa-Ec) of the Museo de Historia Natural de la Universidad de Caldas (localities 17–26).

Number	Department, Municipality	Localities	Latitude, Longitude	Elevation (m)	Dates
1	Caldas, La Dorada	Vereda La Atarraya, near La Miel River	5,72015, -74,72697	178	8/11/2022
2	Caldas, La Dorada	Vereda La Atarraya, Jardín Botánico del Magdalena	5,67694, -74,74417	224	9/11/2022
3	Caldas, Norcasia	Vereda Las Delicias, near the Manso River	5,67261, -74,84481	214	6/04/2022
4	Caldas, Norcasia	Vereda La Estrella, finca El Encanto	5,62775, -74,86806	654	7/04/2022
5	Cundinamarca, Villeta	Vereda Mave	4,94047, -74,45944	1289	15/01/2023
6	Cundinamarca, Villeta	Vereda Cune, Reserva Forestal la Playita	5,04239, -74,50117	1042	17/07/2022
7	Cundinamarca, Villeta	Vereda Cune, Finca Chamorro	5,03258, -74,49281	1044	12/02/2023
8	Cundinamarca, Villeta	Vereda Cune, Finca Choquenzá	5,05314, -74,49375	1271	17/01/2023
9	Cundinamarca, Villeta	Vereda Bagazal	4,98789, -74,48969	868	18/01/2023
10	Cundinamarca, Villeta	Vereda Salitre Blanco	5,05064, -74,49117	1324	12/07/2022
11	Cundinamarca, Villeta	Vereda La Esmeralda	5,05511, -74,54386	1999	17/03/2023
12	Cesar, Jagua de Ibirico	Mina Cerro Largo	9,54533, -73,28578	209	23/09/2021
13	Cesar, El Paso	Mina El Descanso	9,72156, -73,42611	64	17/09/2023
14	Cesar, La Loma	Mina La Loma	9,60972, -73,52089	56	05/03/2021 and 08/07/2021
15	Tolima, Ambalema	Chorrillo	4,43330, -74,80000	273	08/2012 and 11/2012
16	Tolima, Melgar		4,20358, -74,64337	322	04/1962
17	Caldas, Samaná	Vereda Lagunilla	5,60813, -74,94997	866	24/11/2021
18	Caldas, Samaná	Vereda Piedras verdes	5,60736, -74,94446	760	07/11/2021
19	Caldas, Samaná	Vereda La Reforma	5,58329, -74,95034	884	26/11/2021
20	Caldas, Samaná	Parque Nacional Natural Selva de Florencia	5,51642, -75,04292	1478	20/02/2018
21	Huila, Acevedo	Vereda La Ilusion	1,66045, -76,02625	1515	21/10/2021
22	Cundinamarca, La Palma		5,36056, -74,38972	1447	12/09/2018
23	Cundinamarca, Tenjo	Vereda Churunguaro	4,87532, -74,14609	2612	27/09/2018
24	Cundinamarca, Guachetá	Vereda Guachetá Alto	5,38556, -73,68555	2688	22/09/2018
25	Cundinamarca, Sasaima	RFPP Peñas del Aserradero	4,88008, -74,43585	2295	04/10/2018
26	Cundinamarca, La Vega	Vereda San Antonio	4,94875, -74,38367	1372	10/11/2018

To capture bats, we installed 5 nylon mist nets (12.0 m × 2.5 m, and mesh size 36 mm) for five nights at each sampled location. Mist nets were randomly placed and operated between 18:00 and 22:00 hours. We placed bats individually in cotton bags and identified them using taxonomic keys (i.e., Gardner 2008; Díaz et al. 2021). To mitigate possible contamination between samples, the cloth bags used for bat sampling were cleaned and employed only once each night. We collected some specimens from the captured individuals to confirm identifications and deposited them in the Mammals (M) and Ectoparasites (Ec) collections of the Museo de Historia Natural, Universidad de Caldas (MHN-UCa), Colombia.

We manually collected bat flies using entomological tweezers. The collected bat flies were placed in Eppendorf tubes with 70% ethanol to prevent desiccation during transport to the laboratory. For the identification of Streblidae and Nycteribiidae specimens, we used the dichotomous keys and descriptions from Wenzel et al. (1966), Wenzel (1976), Guerrero (1994, 1995, 1998), Autino et al. (1999), and Graciolli (2004). All the collections were conducted under permits granted by the National Authority for Environmental Licenses (ANLA) to the Universidad de Caldas, as stipulated in Resolution 02497 of 2018, and to the National University of Colombia through Resolution 01435 of September 3, 2018, and to the Universidad de Caldas as stipulated in resolution 854 of 20 May 2019, modified by resolution 519 of 3 March 2022.

### ﻿Review of records in the literature and biological collections

To compile additional records within the study area, we reviewed and identified bat and associated bat flies from different locations in the Magdalena River basin encompassing the departments of Caldas, Cundinamarca, Huila, Santander, and Tolima, deposited in the MHN-UCa-Ec collection. Additionally, we conducted searches for studies on ectoparasitic flies associated with bats in the Magdalena River Basin region. We reviewed the available information retrieved from search engines such as Science Direct, Web of Science, SciELO, Scopus, and Google Scholar, using the keywords ((fly*) OR (flies) AND (Streblidae*) OR (Nycteribiidae) AND (bat*) AND (Colombia*)). The last search was performed in October 2023. We also reviewed references and sources cited in the publications to obtain as much information as possible for creating interaction networks. We considered articles that included records of interactions between bat flies and bats in the Magdalena River Basin region, with no temporal restrictions. This approach enabled us to consolidate a more comprehensive data set for our study. We updated the taxonomic names of bat and bat fly’s species reported in the literature. In the case of the fly reported as Paratrichobiuscf.longicrus by Tamsitt and Fox (1970) we listed these records as *P.longicrus* when constructing the interaction network. Similarly, we considered the records of the bats *Sturniralilium* and *S.parvidens* as part of S.cf.giannae, while *S.lilium* is restricted to the South Cone in Argentina, Brazil, Bolivia, Paraguay, and Uruguay; and *S.parvidens* is restricted to Mexico, Guatemala, Belize, Honduras, El Salvador, Nicaragua and Costa Rica (Mammal Diversity Database, 2023).

### ﻿Diversity, structure, and metrics of bat-fly ectoparasitic network

We analyzed the coexistence of bat fly species and their hosts, using the Kendall correlation, which is suitable for small samples and allows for the observation of negative relationships. We excluded infracommunities reported in only one individual as analysis was not possible in such cases. The analyses were performed using the Bipartite package v. 2.20 (Dormann et al. 2008), and the network graphics were created using the “plotweb” function and the “plotModuleWeb” function from the same package, implemented in R software v. 4.3.2 (R Core Team 2022).

To construct the interaction network between bats and bat flies, we classified the associations as primary, non-primary, or accidental, following the criteria established by Dick (2007). Primary associations are defined as those host species infested by ≥ 5% of the total number of individuals of a species of parasite. Additionally, we reviewed the literature to check if associations that were below 5% had been reported previously and if so, we included them in the network. For the data obtained from the review of the MHN-UCa-Ec, we only considered the associations that have been previously reported in the literature. To evaluate host-ectoparasite interactions in the Magdalena River Basin, we unified the data obtained from the different sources mentioned earlier, constructing a two-dimensional matrix through the quantitative summation of the three data sets (fieldwork, literature, and collection specimens).

We also performed bipartite interaction networks, in which bat and ectoparasite species are represented by nodes, and interacting species are linked by lines, with line width proportional to the frequency of each interaction (Blüthgen et al. 2006). Additionally, we evaluated network properties such as complementary specialization (*H_2_*’), specialization at the species level (*d*’), connectance (*C*), and modularity (*M*) (Dormann et al. 2009; Fortuna et al. 2010; Mello et al. 2016). The complementary specialization index (*H_2_*’) measures both the degree of niche complementarity between species and specialization at the species level (Blüthgen et al. 2006; Blüthgen 2010). This index ranges from 0 (unspecialized network) to 1 (perfectly specialized network). The variation of species-level specialization measures (standardized Kullback-Leibler distance or divergence, *d*’) provides valuable information about the structural properties of a network. The *C* index represents the number of interactions or links observed in the network, between bats and their ectoparasitic flies considering the total number of potential interactions. It takes values from 0 to 1 where 0 indicates that there are no connections and 1 that denotes that most of the nodes in the network interact with each other (Blüthgen et al. 2006). We calculated Modularity (*M*) to identify subgroups of species that are more connected to each other than to the rest of the network (modules) (Fortuna et al. 2010). Modularity ranges from 0 to 1, with a value of 1 indicating a highly modular network and 0 a non-modular network. We use the DIRTLPAwd+ algorithm to compute modularity (Dormann and Strauss 2014; Beckett 2016). In addition, we used a null model to test the significance of specialization (*H2*’) and modularity (M) based on 1000 randomly generated matrices based on a Patefield null model (Dormann et al. 2009). Finally, we standardized modularity by calculating the ZQ score (ZQ), where values greater than 1.96 represent differences from the null model (Carstensen et al. 2016).

## ﻿Results

### ﻿Data collection

**Figure 2.**
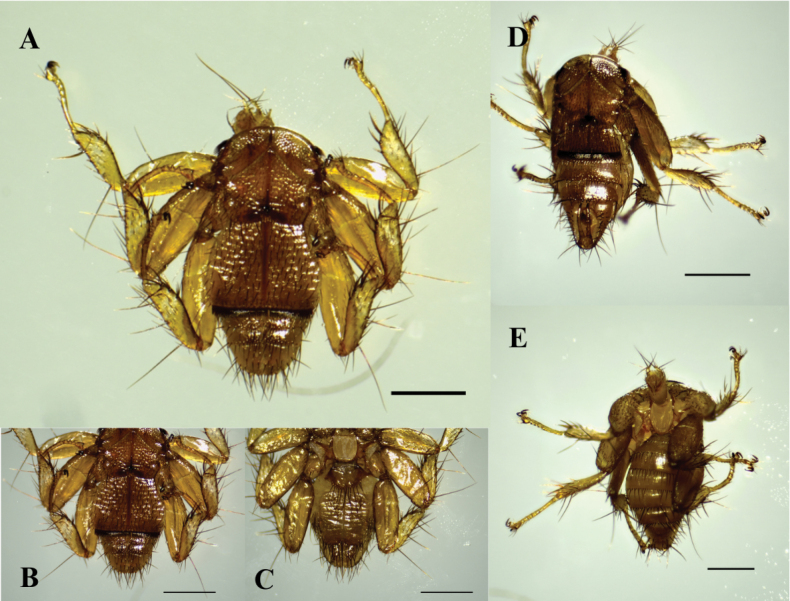
Micrographs of *Basiliajuquiensis* (MHN-UCa-Ec 849), female (**A, B**) ventral view and (**C**) dorsal view; male (**D**) ventral (**F**) dorsal view. Scale bars: 0.5 mm.

During the field work, we captured 376 bats belonging to 31 species, 22 genera, and four families. Of these, 285 bats of 25 species of Phyllostomidae and one species of Noctilionidae carried bat flies. In total we collected 588 bat flies belonging to 23 species, 10 genera and a single family (Streblidae). The most common bat species captured were *Carolliaperspicillata* (*n* = 176), *Carolliabrevicauda* (n = 38), *Glossophagasoricina* (*n* = 23), and *Artibeuslituratus* (*n* = 18). The most abundant species of bat flies were *Trichobiusjoblingi* (*n* = 301) and *Speiseriaambigua* (*n* = 50), mainly associated with species of the genus *Carollia* (Table 2). The literature review added records of 107 bats of eight species of the family Phyllostomidae, of which 51 were being parasitized by 170 bat flies belonging to 14 species of Streblidae. The most frequently reported bat species in the literature were *Carolliaperspicillata* (49), *Artibeusplanirostris* (20), and *Desmodusrotundus* (16). Similarly, the most abundant bat fly’s species recorded in the literature were *T.joblingi* (*n* = 114) and *T.costalimai* (*n* = 82), mainly associated with *Carolliaperspicillata* and *Phyllostomusdiscolor*, respectively (Table 3). The review of specimens housed at the MHN-UCa-Ec added 145 bat flies belonging to 20 species, 11 genera, and two families (Streblidae and Nycteribiidae), linked to 67 bats of 19 species of Phyllostomidae, and two species of Vespertilionidae. *Carolliaperspicillata* (*n* = 23) and *T.joblingi* (*n* = 48) were the most abundant bat and bat fly species (Table 3). For Nycteribiidae, all specimens recorded belong to the genus *Basilia*. The male specimens of *Basilia* deposited at the MHN-UCa-Ec could not be identified to the species level, since the majority of identification keys available correspond to females. We identified *Basiliajuquiensis* males because they were collected with females (Fig. 2), and the latter are characterized by having the sternite almost twice as long as it is wide, sternite III covered by sternite II, and sternite VI divided longitudinally.

**Table 2.** Bat-fly interactions including bat species, number of infested individuals, their respective bat flies, abundance, prevalence of these relationships in the study area. The localities where the associations were documented correspond to Table 1. M (Mammals) and Ec (Ectoparasite): museum vouchers of bat and fly’s specimens deposited at the MHN-UCa.

Bat species	*n*	No. of infested bats	Bat flies	*n*	Prevalence %	Voucher	Locality
**Emballonuridae**							
*Saccopteryxleptura*	2	0	0	0	0	M-3969, M-4223	1, 3
*Rhynchonycterisnaso*	3	0	0	0	0	M-3970, 3971, 4222	2, 3
**Molossidae**							
*Cynomopsgreenhalli*	1	0	0	0	0	M-4221	2
*Molossopsgriseiventer*	1	0	0	0	0	M-4220	2
*Molossusmolossus*	2	0	0	0	0	M-4357, 4358	9
**Noctilionidae**			**Streblidae**				
*Noctilioalbiventris*	4	4	*Paradyschiraparvuloides*	5	100	Ec-1364, 1390, 1392	14
		1	*Trichobiusjoblingi*	2	25	Ec-1384	14
**Phyllostomidae**							
*Carolliabrevicauda*	38	2	*Speiseriaambigua*	3	5.26	Ec-1019, 1326	4, 14
		1	*Streblaguajiro*	1	2.63	M-4341; Ec-1700	5
		19	*Trichobiusjoblingi*	40	50	M-4339-4341; Ec-936, 941-944, 1023, 1025, 1217, 1317, 1328, 1632, 1687, 1689, 1691, 1694, 1701, 1758, 1767	2, 3, 5, 7, 8, 10, 14
		2	*Trichobiusuniformis*	7	5.26	M-4105-4106; Ec-1629-1630	10
*Carolliacastanea*	9	3	*Speiseriaambigua*	3	33.33	M-3974; Ec-934, 1015, 1538	3, 4
		6	*Trichobiusjoblingi*	6	66.67	M-4117; Ec-935,1022, 1190, 1220, 1227, 1684	2, 3, 4, 6
*Carolliaperspicillata*	176	38	*Speiseriaambigua*	45	21.59	M-4108, 4109, 4340; Ec-937,938, 950, 1011, 1013, 1021, 1369, 1373, 1376, 1382, 1386, 1396, 1398, 1400, 1408, 1412, 1415, 1484, 1490, 1492, 1497, 1501, 1504, 1506, 1512, 1515, 1528, 1531, 1541, 1546, 1551, 1555, 1637,1640,1649, 1654, 1658, 1755	3, 4, 5, 12, 13, 10
		1	*Streblaguajiro*	1	0.57	M-4343; Ec-1702	4
		1	*Streblachristinae*	1	**0.57**	Ec-1381	14
		5	*Streblahertigi*	5	2.84	M- 4109; Ec-1014, 1026, 1647, 1505, 1545	4, 13
		2	*Trichobiuscostalimai*	11	1.14	Ec-1332, 1403	14
		1	*Trichobioidesperspicillatus*	1	0.57	Ec-1404	14
		97	*Trichobiusjoblingi*	217	55.11	M-4102-4104, 4107-4109, 4338, 4340; Ec-939, 940, 949, 951, 1024, 1027, 1083, 1212, 1215, 1218, 1222, 1224, 1314, 1322, 1323, 1329, 1331, 1333, 1336, 1341, 1343, 1370-1372, 1374, 1375, 1378, 1380, 1385, 1387, 1388, 1394, 1397, 1405, 1406, 1410, 1413, 1414, 1416, 1417, 1419, 1480, 1482, 1489, 1491, 1493, 1495, 1496, 1498-1500, 1502, 1503, 1509, 1510, 1513, 1516, 1521, 1522, 1526, 1527, 1530, 1532, 1533, 1540, 1542, 1543, 1544, 1547, 1548, 1550, 1554, 1623, 1624, 1628, 1633, 1635, 1636, 1638, 1639, 1641-1644, 1648, 1652, 1653, 1657, 1686, 1688, 1690, 1692, 1693, 1696, 1756, 1757	1, 2, 3, 4, 5, 7, 10, 12, 13, 14
		1	*Trichobiuspersimilis*	3	0.57	Ec-1020	4
		1	*Trichobiusuniformis*	1	0.57	Ec-1340	14
*Anouracadenai*	1	1	*Trichobiusjoblingi*	2	100	M-4355; Ec-1629	5
*Anouraluismanueli*	3	1	*Anastreblamodestini*	2	33.33	M-4428-4430; Ec-1794	11
*Glossophagasoricina*	23	2	*Paraeuctenoideslongipes*	2	8.70	Ec-1487, 1553	14
		1	*Speiseriaambigua*	1	4.35	Ec-1549	14
		9	*Trichobiusjoblingi*	14	39.13	Ec-1321, 1338, 1339, 1342, 1399, 1483, 1486, 1523, 1529	14
		9	*Trichobiusuniformis*	19	39.13	M-4096; Ec-1334, 1335, 1337, 1401, 1402, 1552, 1646, 1655, 1660	10
*Trinycterisnicefori*	1	1	*Streblaalvarezi*	3	100	M-4255; Ec-1197	1
*Lonchorrhinaaurita*	1	1	*Speiseriaambigua*	1	100	M-3973; Ec-957	3
		1	*Trichobius* sp.	4	100	Ec-958	3
*Micronycterismegalotis*	1	0	0	0	0	M-4094	10
*Micronycterismicrotis*	2	0	0	0	0	M-4254, 4356	3, 11
*Lophostomanicaraguae*	3	1	*Streblatonatidae*	3	33.33	M-4236, 4238,1198	1
		2	*Trichobiusmendezi*	4	66.67	Ec-1196, 1202	1
*Lophostomasilvicola*	8	1	*Mastopteraguimaraesi*	1	12.50	Ec-1324	14
		5	*Trichobiusjoblingi*	30	62.50	Ec-1391, 1409, 1507, 1508, 1511	14
*Phyllodermastenops*	1	1	*Streblachristinae*	3	100	M-3972; Ec-948	3
*Phyllostomusdiscolor*	18	1	*Streblahertigi*	1	5.56	Ec-1683	10
		1	*Paratrichobiuslongicrus*	1	5.56	Ec-1315	14
		8	*Trichobioidesperspicillatus*	26	44.44	M-4113,4114; Ec-1669, 1671, 1674-1676, 1678, 1680, 1682	10
		7	*Trichobiuscostalimai*	29	38.89	Ec-1670, 1672, 1673, 1677, 1679, 1681, 1188	10
*Phyllostomushastatus*	12	4	*Mastopteraguimaraesi*	14	33.33	Ec-1194, 1770, 1771, 1792	1, 9
		4	*Streblahertigi*	8	33.33	Ec-1760-1762, 1791	5, 9
		4	*Trichobiusdugesioides*	2	33.33	M-4334; Ec-1195, 1763, 1769, 1772	1, 5, 9
*Artibeusaequatorialis*	8	2	*Aspidopteraphyllostomatis*	2	25	M-3987,4251; Ec-945, 1205, 1206, 1666	6
		4	*Megistopodaaranea*	6	50	M-3989, 4111; Ec-959, 1662, 1665	3, 6, 14
		1	*Trichobiusjoblingi*	1	12.50	Ec-1316-1319	14
*Artibeuslituratus*	18	5	*Paratrichobiuslongicrus*	13	27.78	M-4099-4100-4336; Ec-1488, 1634, 1645, 1661, 1698	8, 10, 14
		2	*Trichobiusjoblingi*	3	11.11	Ec-1383,1656	10, 14
*Dermanuraanderseni*	7	0	0	0	0	M-3979, 3980, 4243, 4248, 4252, 4253, 4258	1, 2, 3
*Mesophyllamacconnelli*	1	0	0	0	0	M-3981	3
*Platyrrhinushelleri*	4	0	0	0	0	M-3984, 3985, 4244, 4249	1, 2, 3
Sturniracf.giannae	21	4	*Aspidopteradelatorrei*	10	30.77	M-4097, 4098, 4353; Ec-1192,1204,1219, 1225, 1226, 1228, 1622, 1651, 1764	1, 2, 10
		20	*Megistopodaproxima*	26	95.23	M-4097, 4098, 4115, 4116, 4241,4352; Ec-1193, 1395, 1517, 1518, 1524, 1534-1536, 1539, 1621, 1650, 1667, 1685, 1695, 1697, 1765, 1766, 1768, 1793	1, 6, 7, 8, 10, 11, 14
*Sturniraluisi*	3	1	*Aspidopteradelatorrei*	2	33.33	M-3978; Ec-961	3,
		1	*Megistopodaproxima*	3	33.30	M-3977, 4250; Ec-952, 1204	2, 3
*Urodermaconvexum*	2	0	0	0	0	M-3986, 4240	1, 3
*Vampyressathyone*	2	0	0	0	0	M-3982, 3983	3

**Table 3.** Bat species with associated bat flies recorded in the literature and museum specimens at the MHN-UCa-Ec. The *n* of infested bats is shown only for records in the literature. The localities where the associations were documented correspond to Table 1.

Bat species (n- infested bats)	Bat fly species	*n*	Reference/Voucher	Locality
**Phyllostomidae**	**Streblidae**			
*Carolliabrevicauda* (3)	*Mastopteraminuta*	1	Tarquino-Carbonell et al. 2015	15
	*Streblaguajiro*	1	Tarquino-Carbonell et al. 2015	15
	*Trichobiusjoblingi*	14	Tarquino-Carbonell et al. 2015/ Ec-847, 866, 869	15
*Carolliacastanea* (4)	*Speiseriaambigua*	1	Ec-853	19
	*Trichobiusjoblingi*	3	Ec-531	19
	*Trichobiuspersimilis*	3	Ec-854, 860	17, 19
*Carolliaperspicillata* (23)	*Megistopodaproxima*	4	Ec-845	18
	*Paratrichobiuslongicrus*	6	Ec-865, 868	17
	*Speiseriaambigua*	19	Tamsitt and Fox 1970; Tarquino-Carbonell et al. 2015/ Ec-529, 533, 856	15, 16, 17, 18
	*Streblaguajiro*	2	Tamsitt and Fox 1970; Tarquino-Carbonell et al. 2015	15, 16
	*Streblahertigi*	2	Ec-858	17
	*Trichobiusjoblingi*	144	Tamsitt and Fox 1970; Tarquino-Carbonell et al. 2015/ Ec-97, 527, 528, 534, 535, 844, 846, 848, 851, 855, 857, 859, 864, 867, 871	15, 16, 17, 18, 19, 20
	*Trichobiustiptoni*	3	Ec-1821	22
*Desmodusrotundus*	*Trichobiusparasiticus*	53	Tarquino-Carbonell et al. 2015	15
*Anouraaequatoris* (1)	*Exastiniondecepticum*	2	Ec-1825	25
*Anoura caudiferа* (1)	*Anastreblacaudifera*	1	Ec-1822	24
	*Anastreblamattadeni*	1	Tamsitt and Fox 1970	16
*Anourageoffroyi* (2)	*Exastiniondecepticum*	3	Ec-1816	23
*Anoura* sp. (3)	*Exastiniondecepticum*	1	Ec-1831	26
	*Anastreblacaudifera*	1	Ec-1829	26
*Anouraperuana*	*Exastinionclovisi*	1	Tamsitt and Fox 1970	16
*Choeroniscus* sp. (1)	*Streblahertigi*	1	Ec-530	20
*Glossophagasoricina*	*Trichobiusdugesii*	1	Tamsitt and Fox 1970	16
	*Trichobiusuniformis*	1	Tarquino-Carbonell et al. 2015	15
*Lonchophyllarobusta* (1)	*Trichobiuslonchophyllae*	1	Ec-1817	22
*Lophostomanicaraguae* (5)	*Mastopteraminuta*	2	Ec-861, 863	17
	*Trichobiusaffinis*	3	Ec-862, 870	17, 18
	*Trichobiuspersimilis*	1	Ec-852	17
*Phyllodermastenops* (1)	*Streblachristinae*	6	Ec-689	18
*Phyllostomusdiscolor* (1)	*Trichobiuscostalimai*	82	Tamsitt and Fox 1970	16
	*Trichobioidesperspicillatus*	73	Tamsitt and Fox 1970/ Ec-1819	16, 22
	*Streblaconsocius*	1	Tamsitt and Fox, 970	16
	*Streblahertigi*	7	Tamsitt and Fox 1970	16
*Phyllostomushastatus*	*Trichobiuslongipes*	3	Tarquino-Carbonell et al. 2015	15
	*Mastopteraminuta*	35	Tarquino-Carbonell et al. 2015	15
*Artibeusaequatorialis*	*Megistopodaaranea*	2	Tamsitt and Fox 1970	16
	*Paratrichobiuslongicrus*	1	Tamsitt and Fox 1970	16
*Artibeuslituratus*	*Aspidopteraphyllostomatis*	2	Tarquino-Carbonell et al. 2015	15
	*Megistopodaaranea*	2	Tarquino-Carbonell et al. 2015	15
	Paratrichobiuscf.longicrus	23	Tamsitt and Fox 1970	16
*Artibeusplanirostris*	*Megistopodaaranea*	3	Tarquino-Carbonell et al. 2015	15
*Artibeus* sp. (2)	*Megistopodaaranea*	2	Ec-1836	26
	*Aspidopteraphyllostomatis*	1	Ec-1837	26
*Enchisteneshartii* (1)	*Paratrichobiussanchezi*	1	Ec-100	20
*Platyrrhinusvittatus* (1)	*Paratrichobiuslongicrus*	1	Ec-75	20
*Sturnirabogotensis* (1)	*Megistopodaproxima*	1	Ec-73	20
	*Trichobiuspetersoni*	3	Ec-1821	22
*Sturniraerythromos* (2)	*Trichobiuspetersoni*	3	Ec-1817, 1820	22
Sturniracf.giannae (7)	*Aspidopteradelatorrei*	3	Ec-685, 688, 751	21
	*Megistopodaproxima*	20	Tamsitt and Fox 1970; Tarquino-Carbonell et al. 2015/ Ec-526, 532, 536, 687, 1820	15, 16, 18, 20, 22
*Sturniraludovici* (2)	*Megistopodaproxima*	3	Ec-1823	25
	*Trichobiuspetersoni*	2	Ec-1824	25
**Vespertilionidae**	**Nycteribiidae**			
*Myotiskeaysi* (1)	*Basilia* sp.	1	Ec-752	21
*Myotisriparius* (2)	*Basilia* sp.	2	Ec-74	20
	*Basiliajuquiensis*	4	Ec-849	17

### ﻿Structure and metrics of the bat-fly ectoparasitic network

The bat-fly bat interaction network for the Magdalena River Basin exhibited high specialization (*H_2_*’ = 0.74) and low connectance (*C* = 0.06). Likewise, the specialization index by bat fly species indicating that 23.68% of species were highly specialized (*d*’ = 0.936–1). The results obtained from the reciprocal specialization index at the species level (*d*’) revealed that the species *Anastreblamodestini*, *Exastinionclovisi*, *Paratrichobiussanchezi*, *Streblaalvarezi*, *S.christinae*, *Trichobiuslonchophyllae*, and *T.parasiticus* each had a value of 1, indicating high reciprocal specialization. These species were followed by *Exastiniondecepticum* (*d*’ = 0.97) and *Paradyschiraparvuloides* (*d*’ = 0.93). In contrast, the species with the lowest values in the specialization index were *Streblaguajiro* (*d*’ = 0.11) and *S.consocius* (*d*´ = 0.11), *S.hertigi* (*d*´ = 0.19), and *Speiseriaambigua* (*d*’ = 0.28), suggesting lower specialization compared to the aforementioned species (Suppl. material 1: table S1).

**Figure 3.**
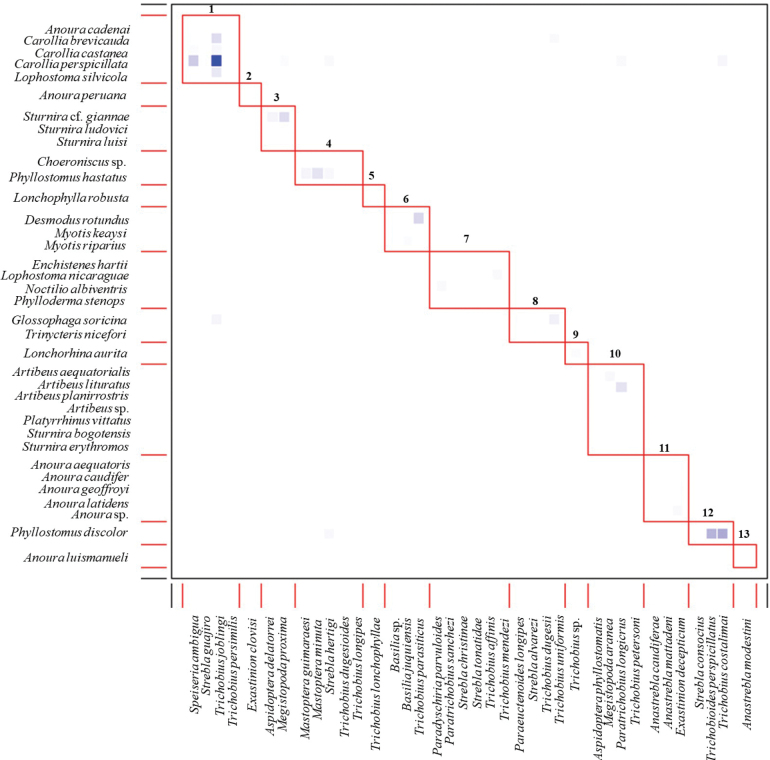
Modularity network generates in the bipartite bat-bat fly network in the inter-Andean Magdalena River basin of Colombia. The intensity of the blue box colors indicates the strength (intensity or frequency) of the interaction, due to the number of fly individuals involved.

Of the 38 species of bat flies included in our interaction network, 19 were associated with a single bat species: *A.mattadeni*, *A.modestini*, *B.fuquiensis*, *E.clovisi*, *P.parvuloides*, *Paraeuctenoideslongipes*, *Paratrichobiussanchezi*, *S.alvarezi*, *S.christinae*, *S.consocius*, *S.tonatidae*, *Trichobioidesperspicillatus*, *Trichobiusaffinis*, *T.dugesii*, *T.dugesioides*, *T.lonchophyllae*, *T.longipes*, *T.mendezi*, and *T.parasiticus*. The bat fly species with the greatest number of interactions were *T.joblingi* (9) and *Megistopodaproxima* (6). The bat species with the most associations were *C.perspicillata* (8), *A.lituratus* (5), and *Lophostomanicaraguae*, *Phyllostomusdiscolor* and *P.hastatus* (5). Additionally, 16 of the 37 bat species used to create the interaction network were associated with only one bat fly species (Suppl. material 1: fig S1). Moreover, we recorded high modularity for the interaction network (*M* = 0.64), forming 13 modules of related species. Most modules exhibited medium (the fly interacts with the host bat species with a noticeable, but not constant, frequency) to low (the fly interacts with the host bat species on rare occasions or under specific conditions) interaction strength (Fig. 3), except for the first module, which showed high interaction strength (the bat fly interacts with the host species frequently and regularly). This indicates a strong dependence of the fly on that particular bat for its survival and reproduction, specifically between *C.perspicillata* and *T.joblingi*. Most modules were formed by phylogenetically similar host, either from the same genus (modules 3 and 11 in Fig. 3) or from the same family (module 1 and 10 of Fig. 3). Comparisons with null models showed differences (ZQ = 83.51) indicating that the observed indices result from ecological processes rather than random chance. Furthermore, the comparison between the observed *H_2_*’ value (0.74) and the null model values revealed significant differences (p-value = 0.00) (Suppl. material 1: fig. S2).

**Figure 4.**
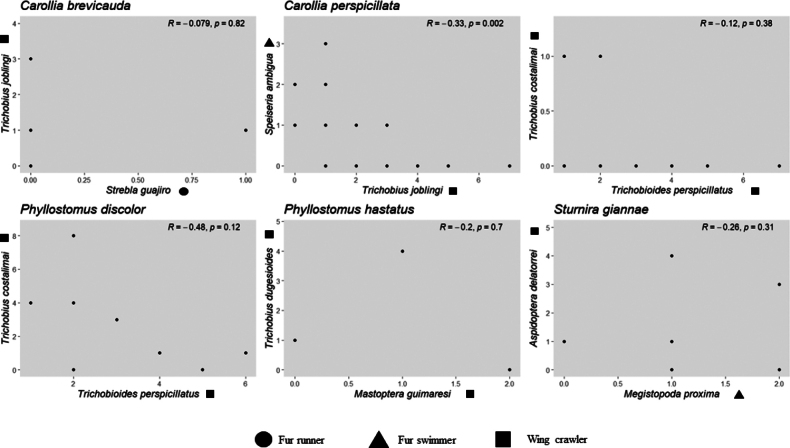
Scatterplots of densities for all bat fly species pairs occurring on their respective host bat species along the inter-Andean Magdalena River basin of Colombia.

**Figure 5.**
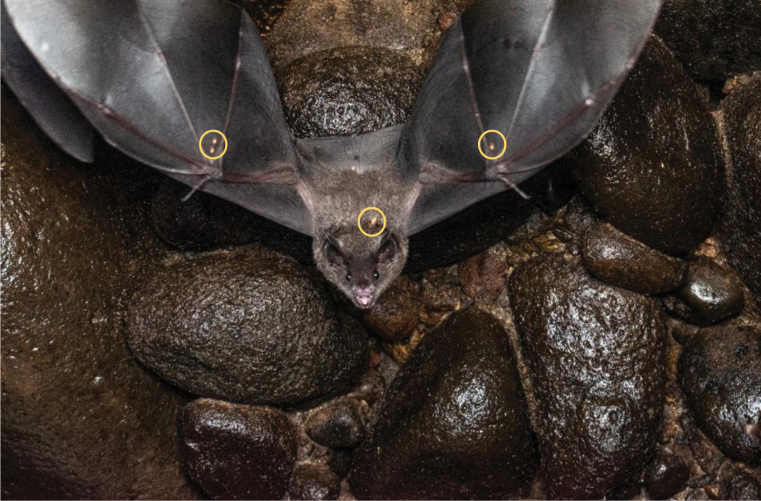
*Carolliaperspicillata* parasitized by two species of bat flies belonging to two different ecomorphological groups *Trichobiusjoblingi* (wing crawler) and *Speiseriaambigua* (fur runner). Photograph: Carlos González Salazar.

We identified infracommunities associated with six bat species within Phyllostomidae: *A.aequatorialis*, *C.brevicauda*, *C.perspicillata*, *P.discolor*, *P.hastatus*, and *S.giannae* (Table 4). However, co-occurrence analyses of bat-bat fly species could be conducted for only five bat species (Table 4) due to the limited sample size of *A.aequatorialis*. The interaction between *T.joblingi* and *S.ambigua* on *C.perspicillata* showed negative interactions (z = -2.867, -0.311, p-value < 0.05), while the remaining five pairs showed positive or null density correlations (Table 4; Fig. 4). Notably, *Carolliaperspicillata* was parasitized by two species of bat flies belonging to two different ecomorphological groups: wing crawlers and fur runners (Fig. 5).

**Table 4.** Co-occurrence analyses (Kendall correlation) of bat fly species on their specific host bat species. Bold p-value indicates statistical significance (p < 0.05).

Infracommunity	*n*	z-value	tau	p-value
***Carolliabrevicauda***	23			
*Streblaguajiro* + *Trichobiusjoblingi*	1	-1.574	-0.537	0.12
***Carolliaperspicillata***	115			
*Speiseriaambigua + T.joblingi*	25	-2.867	-0.311	**0.002**
*S.hertigi + T.joblingi*	3	-0.872	-0.122	0.382
***Phyllostomusdiscolor***	9			
*Trichobiuscostalimai + Trichobioidesperspicillatus*	6	-1.562	-0.48	0.118
***Phyllostomushastatus***	9			
*Mastopteraguimaraesi* + *Trichobiusdugesioides*	1	-0.382	-0.2	0.702
***Sturniragiannae***	11			
*Aspidopteradelatorrei* + *Megistopodaproxima*	2	-1.022	-0.259	0.306

## ﻿Discussion

This study extends knowledge of bat flies interactions in the Magdalena River basin and in Colombia, as we included the largest number of species of both bats (42 spp.) and bat flies (35 spp.) in the inter-Andean valleys of the country. The families Phyllostomidae (Chiroptera) and Streblidae (Diptera) were the most diverse, which was expected due to the positive correlation with capture methods (mist nets) in Neotropical regions (Dick and Gettinger 2005; Patterson et al. 2008). In contrast, bat flies within Nycteribiidae are rarely represented due to the low number of captures of their host bat families such as Vespertilio­nidae. In this context, the diversity of Nycteribiidae is underestimated and re­presents a gap to be filled in the future. Similarly, the presence of nycteriibids in other poorly represented bat families such as Emballonuridae and Molossidae should be assessed. These families have low capture rates with mist nets due to their elusive behavior and foraging activity between and above the forest canopy (Bonaccorso 1979; Muñoz Arango 2001; Gardner 2008; Tarquino-Carbonell et al. 2015). New records or associations are therefore expected. For example, of the 35 species of bat flies recorded in the Magdalena River basin, *Basiliajuquiensis* associated with the bat *Myotisriparius* from Samaná, Caldas (Fig. 2) is a new record for Colombia. This fly was previously known only from Brazil and Venezuela, associated with *Myotisnigricans* and *M.riparius* (Graciolli 2004).

Our results indicate a predominance of certain bat fly species, such as *Trichobiusjoblingi*, which is the most common ectoparasite of bats in the Magdalena River basin. This dominance is largely attributed to its close association with bats of the genus *Carollia*, particularly *C.perspicillata* which is considered its main host (Wenzel et al. 1966; Fritz 1983). Given the high abundance of *C.perspicillata* in the study area, the prevalence of *T.joblingi* was anticipated. Associations with other bat species are likely, in some cases, accidental (Wenzel 1976; Komeno and Linhares 1999; Barbier and Graciolli 2016).

The Magdalena River basin is experiencing high levels of deforestation due to intense human activity, which has significantly altered the original landscape (Restrepo and Syvitski 2006). Consequently, the elevated prevalence of bat flies in this region may correlate with the intensity of landscape transformation. While this study focused on a specific region, the prevalence of bat fly observed aligns with findings from studies conducted in urban areas of the tropical region (Zarazúa-Carbajal et al. 2016; Hernández-Martínez et al. 2018; Urbieta et al. 2021). In these urban areas, the responses of bat ectoparasites to habitat loss and fragmentation tend to be host- and parasite-specific (Pilosof et al. 2012; Bolívar-Cimé et al. 2018; Hiller et al. 2020, 2021; Eriksson et al. 2023). For instance, Mello et al. (2023) found that certain bat fly species, including *Megistopodaproxima*, *Streblaguajiro*, and *Trichobiusjoblingi* are more prevalent in deforested areas. Generally, disturbed and fragmented sites lead to a reduction in the availability of high-quality roosts, which can result in the overcrowding of bats and the formation of multi-species colonies (Brändel et al. 2020; Kelm et al. 2021). Under such conditions, ectoparasites may switch primary hosts, resulting in a loss of specialization in their interactions and facilitating horizontal transfer (Lewis 1995; Dick and Patterson 2006; [Bibr B72][Bibr B35]; Saldaña-Vázquez et al. 2019).

Additionally, our results indicate medium modularity in the interactions between bats and bat flies, which correlates with medium specialization and low connectivity (Blüthgen et al. 2006; Durán et al. 2018). The identified subgroups reflect the niche differentiation of bat flies among their hosts (Blüthgen et al. 2006). Most modules are formed by the phylogenetic preference of different bat fly species for specific bat species. For instance, we observed that species of the genus *Anoura* (*Anouraaequatoris*, *A. caudiferа*, *A.geoffroyi*, *A.latidens*, and *Anoura* sp.) were parasitized by *Anastreblacaudifera* and *Exastiniondecepticum* (Fig. 3). It is likely that the parasites select phylogenetically related hosts due to their phenotypic similarities (Wiens et al. 2010; Lima et al. 2012). The close evolutionary relationship between bat flies and bats, with the phenotypic similarities among phylogenetically related bats, may serve as a filter for parasite species (Dick and Patterson 2006; Wiens et al. 2010; Urbieta et al. 2021).

The infracommunities and parasite associations identified in this study align with previous findings (Fritz 1983; Dick 2005; Bezerra et al. 2016; Dornelles et al. 2017; Bezerra and Bocchiglieri 2018) and may result from ecomorphological differentiation, where unrelated parasites coexist on the same host and spatially segregate within the host’s body (Dick 2005). In all documented cases, the infracommunities comprised parasite species from different genera and primarily from different ecomorphological groups (Dick 2005; Hiller et al. 2018). In our findings, all the relationships between pairs of bat fly species were negative, with only one pair exhibiting a significantly negative correlation, interpreted as potential competition for limited resources, between *S.ambigua* and *T.joblingi* on *C.perspicillata*. This suggests that while the presence of one bat fly species does not preclude the presence of another, their abundances are negatively correlated (density compensation) (Wenzel et al. 1966; Dick and Patterson 2006; Presley 2007; Tello et al. 2008). Consequently, negative correlations between pairs of species on the same host may serve as a mechanism to maintain ectoparasite populations within the host’s tolerable limit (Komeno and Linhares 1999).

In conclusion, our results indicate that interactions between bats and bat flies may vary based on habitat conservation status, potentially leading to lo­wer specialization in degraded and fragmented landscapes. While our study did not directly assess this relationship, we recommend that future research delve deeper into how habitat conservation influences the specialization of these interactions. Additionally, our results suggest that different species of bat flies can coexist and share the same resource (bats), with their morphological traits likely playing a role in this coexistence. Overall, this study enhances our understanding of bat flies-bats interactions in the Magdalena River basin and expands the known distribution of certain bat fly species within the country.

## ﻿Additional information

### Author ORCIDs

Camila López-Rivera https://orcid.org/0009-0003-5414-7956

Laura Natalia Robayo-Sánchez https://orcid.org/0000-0002-4074-1001

Alejandro Ramírez-Hernández https://orcid.org/0000-0001-8689-5930

Jerson Andrés Cuéllar-Saénz https://orcid.org/0000-0003-2257-8981

Juan Diego Villar https://orcid.org/0009-0006-8820-1908

Jesús Alfredo Cortés-Vecino https://orcid.org/0000-0003-2641-604X

Fredy Arvey Rivera-Páez https://orcid.org/0000-0001-8048-5818

Paula Andrea Ossa-López https://orcid.org/0000-0002-9079-4988

Erika M. Ospina-Pérez https://orcid.org/0000-0001-5784-6216

Jose J. Henao-Osorio https://orcid.org/0000-0002-8618-8539

Alexandra Cardona-Giraldo https://orcid.org/0000-0002-7534-994X

Javier Racero-Casarrubia https://orcid.org/0000-0001-5989-4174

Darwin M. Morales-Martinez https://orcid.org/0000-0001-5786-4107

Marylin Hidalgo https://orcid.org/0000-0001-8960-0616

Héctor E. Ramírez-Chaves https://orcid.org/0000-0002-2454-9482

### Data availability

All of the data that support the findings of this study are available in the main text or Supplementary Information.

## Supplementary materials

Supplementary material 1 Interaction network between bats and bat-flies in the Magdalena River basin region and null models Data type docx https://binary.pensoft.net/file/1210722This dataset is made available under the Open Database License (http://opendatacommons.org/licenses/odbl/1.0/). The Open Database License (ODbL) is a license agreement intended to allow users to freely share, modify, and use this Dataset while maintaining this same freedom for others, provided that the original source and author(s) are credited.Camila López-Rivera, Laura Natalia Robayo-Sánchez, Alejandro Ramírez-Hernández, Jerson Andrés Cuéllar-Saénz, Juan Diego Villar, Jesús Alfredo Cortés-Vecino, Fredy A. Rivera-Páez, Paula Andrea Ossa-López, Erika M. Ospina-Pérez, Jose J. Henao-Osorio, Alexandra Cardona-Giraldo, Javier Racero-Casarrubia, Miguel E. Rodríguez-Posada, Darwin M. Morales-Martinez, Marylin Hidalgo, Héctor E. Ramírez-Chaves
