# Supplementary material for: ﻿Diversity of ectoparasitic bat flies (Diptera, Hippoboscoidea) in inter-Andean valleys: evaluating interactions in the largest inter-Andean basin of Colombia
Source: Zookeys. 2024 Dec 31;1221:377–400. doi: 10.3897/zookeys.1221.127890 (PMC11707520; doi:10.3897/zookeys.1221.127890)
Supplement: Supplementary material 1 — Interaction network between bats and bat-flies in the Magdalena River basin region and null models [file zookeys-1221-377_article-127890__-s001.docx]

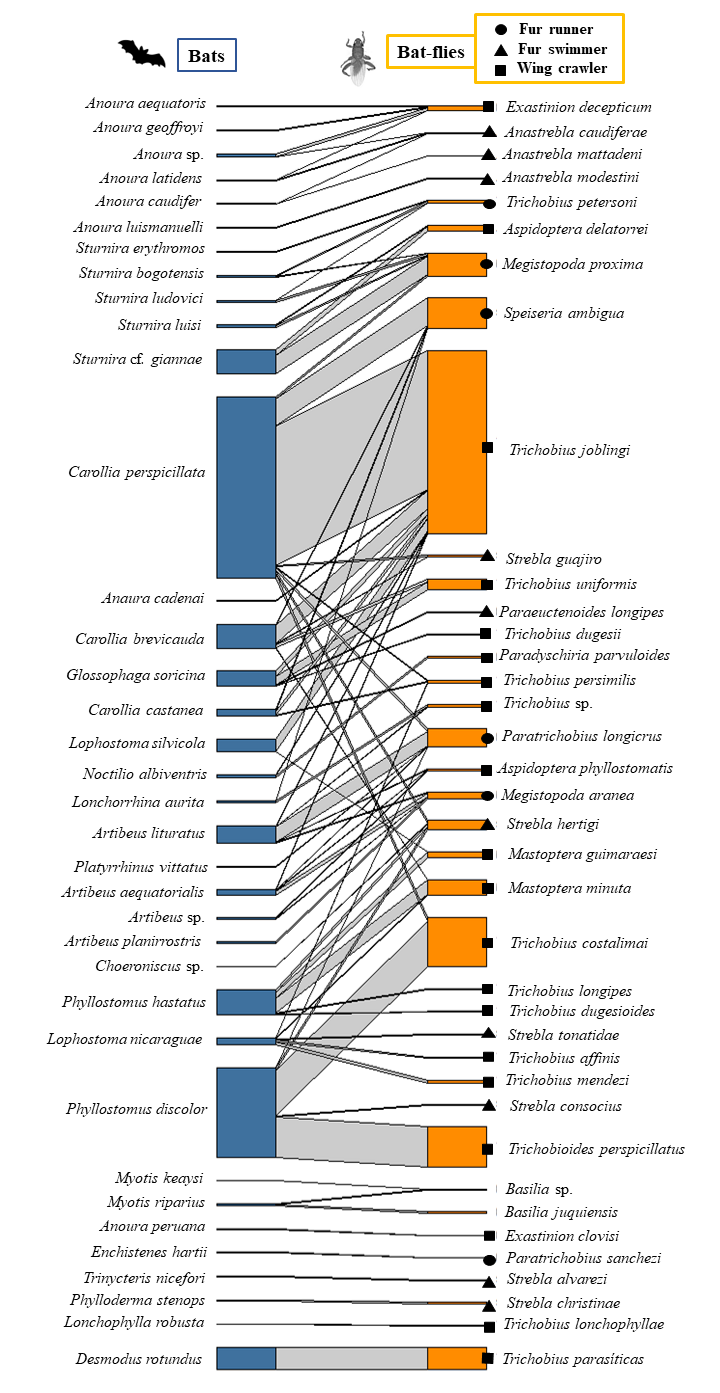


**Supplementary Figure S1.** Bipartite bat-bat fly network in the inter-Andean Magdalena River Basin of Colombia. The size of the blue bar represents the abundance of bats for which bat flies were sampled and the size of the orange bar represents the abundance of bat flies by species observed. The width of the gray lines/bars indicates the frequency of the interactions.


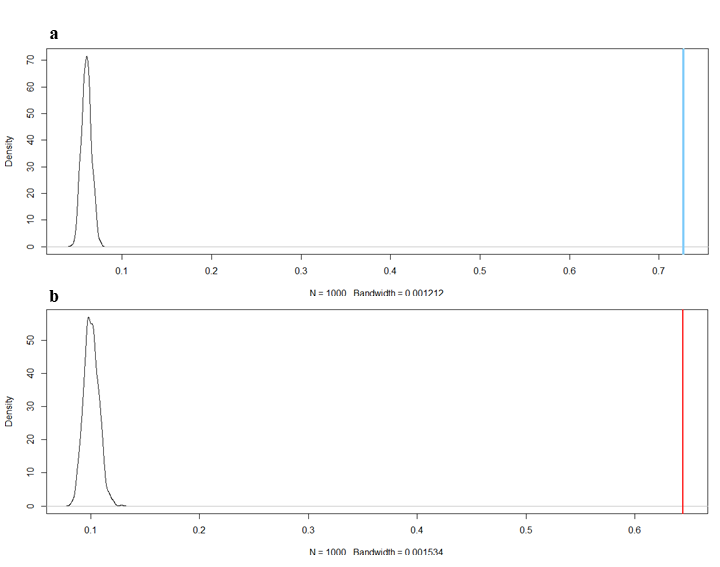


**Supplementary Figure S2.** Plots of the random values (null models) of *M* (a) and *H_2_'* (b) with the observed values. The red and blue lines represent the observed values of *M* and *H_2_'*.

**Supplementary Table S1.** Specialization index of species (d') registered on the ectoparasite bat flies in the inter-Andean Magdalena River Basin of Colombia.

| **Species bat-flie** | **d'** |
| --- | --- |
| *Anastrebla caudiferae* | 0.78 |
| *Anastrebla mattadeni* | 0.88 |
| *Anastrebla modestini* | 1 |
| *Aspidoptera delatorrei* | 0.65 |
| *Aspidoptera phyllostomatis* | 0.55 |
| *Basilia* sp. | 0.86 |
| *Basilia fuquiensis* | 0.92 |
| *Exastinion clovisi* | 1 |
| *Exastinion decepticum* | 0.97 |
| *Mastoptera guimaraesi* | 0.61 |
| *Mastoptera minuta* | 0.77 |
| *Megistopoda aranea* | 0.83 |
| *Megistopoda proxima* | 0.78 |
| *Paradyschiria parvuloides* | 0.93 |
| *Paraeuctenoides longipes* | 0.49 |
| *Paratrichobius longicrus* | 0.71 |
| *Paratrichobius sanchezi* | 1 |
| *Speiseria ambigua* | 0.28 |
| *Strebla alvarezi* | 1 |
| *Strebla christinae* | 1 |
| *Strebla consocius* | 0.11 |
| *Strebla guajiro* | 0.11 |
| *Strebla hertigi* | 0.19 |
| *Strebla tonatidae* | 0.68 |
| *Trichobioides perspicillatus* | 0.66 |
| *Trichobius affinis* | 0.68 |
| *Trichobius costalimai* | 0.56 |
| *Trichobius dugesii* | 0.40 |
| *Trichobius dugesioides* | 0.40 |
| *Trichobius joblingi* | 0.67 |
| *Trichobius lonchophyllae* | 1 |
| *Trichobius longipes* | 0.43 |
| *Trichobius mendezi* | 0.82 |
| *Trichobius parasiticus* | 1 |
| *Trichobius persimilis* | 0.29 |
| *Trichobius petersoni* | 0.92 |
| *Trichobius* sp. | 0.80 |
| *Trichobius uniformis* | 0.72 |
